# Research Progress on the detection methods of porcine reproductive and respiratory syndrome virus

**DOI:** 10.3389/fmicb.2023.1097905

**Published:** 2023-03-09

**Authors:** Jinghua Pan, Mengyi Zeng, Mengmeng Zhao, Liangzong Huang

**Affiliations:** ^1^School of Life Science and Engineering, Foshan University, Foshan, China; ^2^Veterinary Teaching Hospital, Foshan University, Foshan, China

**Keywords:** porcine reproductive and respiratory syndrome, virus isolation, molecular biology, serological assay, metagenomic next-generation sequencing

## Abstract

Porcine reproductive and respiratory syndrome virus (PRRSV) causes clinical syndromes typified as reproductive disorders in sows and respiratory diseases in piglets. PRRSV remains one of the most prevalent pathogens affecting the pig industry, because of its complex infection profile and highly heterogeneous genetic and recombination characteristics. Therefore, a rapid and effective PRRSV detection method is important for the prevention and control of PRRS. With extensive in-depth research on PRRSV detection methods, many detection methods have been improved and promoted. Laboratory methods include techniques based on virus isolation (VI), enzyme-linked immunosorbent assays (ELISA), indirect immunofluorescence assays (IFA), immunoperoxidase monolayer assays (IPMA), polymerase chain reaction (PCR), quantitative real-time PCR (qPCR), digital PCR (dPCR), loop-mediated isothermal amplification (LAMP), recombinase polymerase amplification (RPA), clustered regularly interspaced short palindromic repeats (CRISPR), metagenomic next-generation sequencing (mNGS), and other methods. This study reviews the latest research on improving the main PRRSV detection methods and discusses their advantages and disadvantages.

## 1. Introduction

In the late 1980s, severe disease outbreaks occurred on pig farms in the United States. The disease was characterized by reproductive impairment, decreased growth rate, respiratory symptoms, and increased mortality in sows. Initially, it was not known which pathogen caused the disease, which was originally named “mystery swine disease (MSD)” (Ruedas-Torres et al., 2021). In 1991, etiological studies based on Koch’s postulates showed that the disease was caused by an unknown RNA virus. The virus used in these studies was isolated from Lelystad in the Netherlands (Wensvoort et al., 1991). European scholars proposed the name “porcine reproductive and respiratory syndrome” (PRRS) for the disease in the same year, and the origin of this disease is still being studied (Murtaugh et al., 2010). In 1996, Guo et al. (1996) isolated PRRSV from abortion samples for the first time in China and named one of its strains CH-1a. In 2006, a variant strain of the highly pathogenic porcine reproductive and respiratory syndrome virus (HP-PRRSV) was found on pig farms in the southern provinces of China. In contrast with VR-2332 (PRRSV-2), HP-PRRSV has a discontinuous 30-amino-acid (aa) deletion in the NSP2 coding region (Karniychuk et al., 2010). To date, HP-PRRSV remains one of the major epidemic strains in China. The emergence of a PRRSV NADC30-like strain was first reported in the United States in 2008. Unlike previous HP-PRRSV and classical strains, NADC30-like has a 131aa deletion in the NSP2 coding region (Brockmeier et al., 2012). In 2013, a rapidly spreading NADC30-like strain was identified in China (Zhou et al., 2014). Currently, NADC30-like strains have been found in many provinces in China. The infection rate of the NADC30-like strain is on the rise, and it has become the dominant strain (Deng et al., 2020). In addition, NADC30-like strains with high activity were reported for the first time in Korea in 2014 (Kim et al., 2021). This indicates that NADC30-like strains exist in epidemic form and have significant research value. In 2021, Chen Y. et al. (2021) isolated a novel variant strain of PRRSV (HBap4-2018) and confirmed that this variant belonged to a natural recombinant PRRSV derived from HP-PRRSV and NADC30-like strains. In 2014, the strain NADC34 was first reported in the United States. In contrast to positions 328–427 of the VR–2332 Nsp2, PRRSV NADC34-like is missing 100 aa consecutively at the corresponding positions (van Geelen et al., 2018). The detection rate of NADC34-like as a potentially prevalent strain has been increasing. Some PRRSV strains have a high capacity for genetic variation and a high incidence of recombination, resulting in many variant strains circulating worldwide. Current vaccines are likely to be ineffective against most strains, making immune evasion easier (Guo et al., 2018).

PRRS is one of the most economically destructive viral diseases in the global pig industry. PRRSV has two genotypes: the European type (PRRSV-1), represented by the Lelystad Virus strain, and the North American type (PRRSV-2), represented by strain VR-2332 (Nelsen et al., 1999). Although the onset of disease, clinical symptoms, and genomic architectures of both appear similar, they share only approximately 60% similarity at the nucleotide level (Kappes and Faaberg, 2015). PRRSV is an enveloped RNA virus that belongs to the order Nidovirales and family Arterivirus (Meulenberg et al., 1997). The PRRSV genome is approximately 15 kb long with at least 10 open reading frames (ORFs) (Zhou et al., 2015). The PRRSV genome contains ORF1a, ORF1b, ORF2a, ORF2b, ORF3, ORF4, ORF5, ORF5a, ORF6, and ORF7 when read from the 5′ end to the 3′ end. Among them, ORF2-5 encodes the structural proteins: GP2a, E, GP3, GP4, GP5, and GP5a; ORF6 encodes matrix proteins (M); and ORF7 encodes the viral nucleocapsid protein (N) (Mardassi et al., 1995). The ORF1 protein can be further hydrolyzed into 16 nonstructural proteins, including NSP1α, NSP1β, NSP2-6, NSP-2 N, NSP-2TF, NSP7α, NSP7β, and NSP8-12 (Kappes and Faaberg, 2015). To date, recessive and subclinical infections have become prevalent in many pig farms (Guo et al., 2018). The diversity and complexity of PRRSV compound the difficulties for the pig industry, and no successful treatment has yet been found. Additionally, the clinical symptoms of PRRSV are similar to those of brucellosis, pseudorabies, and porcine parvovirus (PPV), making it difficult to diagnose and identify PRRSV. Establishing rapid, accurate, and efficient detection techniques is, therefore, essential for the timely detection and control of PRRS. In this study, we review the progress in developing PRRSV detection methods, which are essential for the diagnosis and prevention of this disease.

## 2. Virus isolation

The key to isolating the virus is to collect samples of the most recent diseased material with the highest viral content. The samples are then immediately refrigerated and promptly sent for testing. Otherwise, false-negative results are likely to occur during the testing process. The main sites of PRRSV distribution in pigs vary with their age and stage of infection. PRRSV can be isolated from tissue homogenate mixtures in PRRSV-infected pigs, which can include spleens, lungs, lymph nodes, serum, and plasma (Horter et al., 2002). In general, tissue samples with higher detection rates are deep lung effusions and serum (Chen C. et al., 2010), which have higher viral contents than those in other organs.

Generally, virus isolation (VI) is performed using sensitive cell isolation and culture. The cell lines currently used to isolate and proliferate PRRSV are porcine alveolar macrophage (PAM), passenger cells CL262, Marc-145, HS2H cells (derived from Marc-145), and ZMAC cell lines (derived from PAM). The susceptibility of various cell lines to different virulent strains varies. Some strains grow on only one type of cell, while others can grow on multiple types of cells. PAM is particularly susceptible to most strains and produces high levels of virulence, especially for PRRSV-1 (Hu et al., 2011a). In 1991, Wensvoort et al. (1991) removed PAM from the lungs of 6-week-old specific pathogen-free (SPF) pigs and successfully isolated PRRSV for the first time using PAM. Although PAM is suitable for most PRRSVs, its primary cells have a short life cycle and need to be prepared regularly. Furthermore, PAM is susceptible to contamination by other pathogens, and the batch quality of primary cells varies. CL2621 is a proprietary cell line of Boehringer Ingelheim Animal Health (Collins et al., 1992) and is not widely used in most laboratories (Kim et al., 1993). Currently, Marc-145, which is appropriate for PRRSV-2, is the most widely used cell line for VI. Forty-eight hours following the inoculation of Marc-145 cells with PRRSV, typical cytopathic effects (CPE) can be observed. For VI from samples containing Spectrum 1 and Spectrum 8 PRRSV-2 or samples of the same spectrum from lung and serum, the ZMAC cell line had a higher isolation success rate than that of MARC-145 cells (Yim-im et al., 2021). It is worth noting that some classical and highly pathogenic strains of PRRSV should be treated differently when performing the initial isolation. When isolating part of the classical strain, it needs to be adapted to PAM before it can be adapted to MARC-145 cells, and the CPE do not occur easily, whereas the first generation of highly pathogenic virus strains can be adapted to MARC-145 cells and can present CPE (Chou et al., 1998).

VI remains one of the most important pathogen identification techniques. For instance, VI is essential for the amplification, purification, and identification of PRRSV genotypes. PRRSV VI can provide a large resource of virulent strains for studies such as vaccine development and updates or studies on pathogenesis. In addition, the development and validation of new detection methods cannot be done without VI. Although VI is a conventional, classical PRRSV detection method, it has some shortcomings: (i) the operation of VI is time-consuming, tedious, and complex; (ii) it requires highly aseptic operations; (iii) the chance of generating contamination is relatively high; and (iv) the manual labor requirement is large. Therefore, it is not appropriate for large-scale quarantine and quick diagnosis on pig farms.

## 3. Serological detection methods

### 3.1. Enzyme-linked immunosorbent assay

As an antibody detection technique, the enzyme-linked Immunosorbent assay (ELISA) is critical in PRRSV outbreak surveillance and vaccine immunization evaluation. ELISA is a popular method for evaluating clinical material because it is straightforward and highly automated, yielding results in a short amount of time. The main encapsulated antigens commonly used in traditional indirect ELISA are whole viruses and expressed proteins. However, they often have disadvantages, such as low protein purity. In contrast, synthetic peptide antigens have the following advantages: (i) small relative molecular mass, simple structure, and high sensitivity; (ii) the synthetic peptide is a water-soluble compound that can be coated on the enzyme plate, which offers time-saving advantages (Sanjosé et al., 2015); and (iii) ELISA methods using synthetic peptides as antigens for the detection of PRRSV antibodies have a significant price advantage compared with that of commercial ELISA. Peptide-based PRRSV ELISA methods have been developed. For instance, Sun et al. developed an ELISA for the detection of PRRSV antibodies, utilizing artificial peptides as antigens (Sun, 2002). Zhao et al. developed two synthetic M-peptide PRRSV ELISAs for the detection of NADC30-like antibodies (Zhao et al., 2021). Both responded well to the dynamic pattern of PRRSV antibody elongation. Recently, nanobodies have gained increasing attention with the advantage of their small molecular weight and simple genetic engineering, which are good for diagnostic applications (Wang et al., 2016). Duan et al. prepared a nanobody-HRP fusion protein against the anti-PRRSV-N protein and developed the first competitive ELISA for the detection of anti-PRRSV-2 antibodies in pig sera as a probe (Duan et al., 2021). Nanobody-HRP fusion protein against anti-PRRSV-N protein has the advantages of no purification or enzymatic labeling, a stable expression system, simple operation, and easy mass production, which are conducive to a lot of clinical applications.

Antibody determination is the most effective method for evaluating vaccine immunization effectiveness and has a strong persuasive effect on the design of effective immunization programs. Currently, two commonly used commercial PRRSV ELISA kits are the IDEXX HerdChek PRRS Ab X3 (IDEXX Laboratories, Inc., Westbrook, ME, United States.; abbreviated here as IDEXX-ELISA) and the LSIV ET Porcine PRRS/AS-Serum (Laboratoire Service International, Lissieu, France; abbreviated here as LSI-ELISA). Ge et al. conducted a comprehensive comparison of the utility of these two kits in order to identify which is most suitable for HP-PRRSV vaccination programs. This study has shown that IDEXX-ELISA detects antibodies earlier than LSI-ELISA, making it more suitable for the diagnosis of early HP-PRRSV infection (Ge et al., 2019). In addition, the IDEXX-ELISA allows an earlier assessment of whether an immune response has been generated after vaccination. At the later stage of immunization, the level of antibody detected by IDEXX-ELISA was low. This is consistent with neutralizing antibody (NA) levels, indicating low resistance to PRRSV in pigs and the need for enhanced immunity. However, it is still possible to obtain a positive antibody titer with LSI-ELISA during the late immunization period when the NA titer is negative. Thus, the use of LSI-ELISA may increase the chance of wild-type PRRSV infection in pigs.

Although the above-mentioned commercial kits have high accuracy in antibody detection, they cannot effectively differentiate between pigs infected with wild-type PRRSV and vaccinated pigs (Zhang et al., 2021). However, there are many successful ELISAs for confirming early PRRSV infection and differentiating between antibodies induced by wild strains and those induced by vaccines. For example, the indirect ELISA based on recombinant Nsp2 protein developed by Wang et al. (2018) is suitable for the identification of wild-type infected animals from MLV TJM-F92-immunized animals. The blocking ELISA developed by Cong et al. (2013) can be used to differentiate antibodies against live and inactivated PRRSV in pigs. A novel double recognition ELISA based on the nucleocapsid protein was developed by Venteo et al. (2012) for the early detection of PRRSV-1 infection. The ELISA developed by Zhang et al. (2021) has some diagnostic value in detecting antibodies to a live PRRSV infection. Traditionally, serum samples have usually been used for PRRSV antibody testing. However, saliva samples also contain detectable levels of PRRSV-specific antibodies. Croft et al. (2020) developed a commercial ELISA for the detection of salivary antibodies to PRRSV. Saliva sampling is less invasive and more convenient for livestock. Moreover, it can be used in cases of low prevalence or when large-scale pig farms need to be sampled regularly over a long period of time. However, this method is not applicable when there is a weak positive reaction (Table 1).

**Table 1:** Comparison of different PRRSV ELISA methods.

Serial no.	Types of detection method	Protein of target	Application	References
1	A peptide-based ELISA	N	The detection of PRRSV antibodies using synthetic peptides as antigens.	Sun (2002)
		M	Detecting antibodies against NADC30-like PRRSV	Zhao et al. (2021)
2	A nanobody -based competitive ELISA	N	Detecting Antibodies against PRRSV-2	Duan et al. (2021)
3	An indirect ELISA	Nsp2	Differentiating animals infected with wild-type strains from those immunized with MLV TJM-F92.	Wang et al. (2018)
4	A blocking ELISA	Nsp9	Differentiating antibodies against live and inactivated PRRSV	Cong et al. (2013)
5	A double recognition ELISA	N	The early detection of PRRSV-1 infection	Venteo et al. (2012)
6	A commercial oral fluid antibody ELISA	/	Used in cases of low prevalence or where a significant number of animals need to be sampled over a long period of time	Croft et al. (2020)

### 3.2. Indirect immunofluorescent assay

The indirect immunofluorescent assay (IFA) is a method that detects antibodies to or antigens of PRRSV. It is a crucial method for identifying PRRSV because it helps to retain the virus’s structural integrity and the normal conformation of the viral proteins. Positive and negative samples of IFA produce large differences in signal intensity, which can avoid unnecessary errors. The cell reaction plates used during the IFA operation can be stored for a long time at −20°C, which facilitates testing at any time. The assay results can be stored at 4°C for a long duration after observation by fluorescence microscopy, which is beneficial for retaining results. Yoon et al. (1992) developed and standardized an IFA for the detection and quantification of antibodies to PRRSV. Hu (2021) developed an IFA with monoclonal 5B4 cell supernatant against the PRRSV N protein as the primary antibody and goat anti-mouse IgG-FITC fluorescent antibody as the secondary antibody. However, the IFA is not suitable for large-scale routine testing owing to the disadvantage of requiring replication of the virus. In addition, the number of confounding factors in the IFA reaction is high, and the detection results tend to produce non-specific staining.

Monoclonal antibodies (mAbs) are a good choice for specific detection of PRRSV because of their specificity against only a particular antigenic determinant cluster and their ability to stably produce antibodies indefinitely. In recent years, a PRRSV mAb has been successfully produced, and many researchers have prepared PRRSV mAbs targeting a certain structural or non-structural protein found in different types of PRRSV. The nonstructural proteins include NSP2 (Yan et al., 2007; Wang et al., 2008; Hu et al., 2009; Li et al., 2013; Wang H. et al., 2017), NSP7 (Wang F.-X. et al., 2017), NSP9 (Wu et al., 2012; Zhao et al., 2016), NSP10 (Zhang Z. et al., 2017), and NSP12 (Bi et al., 2017); the structural proteins include GP2 (Liu et al., 2006), GP3 (Sun et al., 2013; Liang et al., 2021), GP4 (Guo et al., n.d.; Liu et al., 2019), GP5 (Zhou et al., 2005a,b; Ma et al., 2006, 2007, 2008; Cai et al., 2009; Wang et al., 2009, 2012; Zhong et al., 2011; Tang et al., 2013; Li et al., 2016, 2019; Huang et al., 2019; Yang et al., 2020; Qin et al., 2021; Young et al., 2021; Wang F. et al., 2022; Supplementary Table S1). Some mAbs have been commercialized and are widely used (Supplementary Table S2). When an organism is infected by a virus, the M and GP5 proteins induce the organism to produce antibodies that can neutralize the proteins, which has a protective effect on the organism. Therefore, there are many reports of mAbs against these two proteins (Hu, 2021). In Zhang et al. (2020) developed an mAb based on the anti-CD163 SRCR5-6 region, which has the effect of blocking PRRSV. This study can provide ideas for future research on how to treat PRRSV.

### 3.3. Immunoperoxidase monolayer cell assay

The basic principle of the immunoperoxidase monolayer cell assay (IPMA) is antigen–antibody specific binding, which can be used to detect PRRSV antibodies and needs to be performed on sensitive monolayers of cells, such as Marc-145. It has the advantages of good sensitivity, repeatability, and specificity (Gao, 2020). The test results can be observed and interpreted using an ordinary optical microscope, and the cost of reagents and consumables required for the experiment is low, making it especially suitable for use in grassroots units. In addition, as with IFA, IPMA cell plates can be prepared in advance and stored under cold-chain conditions for long periods of time. Tan et al. (2006) established IPMA to detect PRRSV serum antibodies accurately, especially for PRRSV-2, and developed a PRRSV-IPMA antibody detection kit. Li et al. (2020) used PRRSV multiplex serum as the primary antibody to establish a naked-eye observable IPMA. However, the PRRSV-IPMA also has the disadvantage that it is subject to individual subjective factors in the determination of results. Therefore, specialized operators need to be trained and experience needs to be accumulated. In addition, when the PRRSV content is too low to produce a specific color reaction, the positive detection rate of the sample is slightly lower than that of the polymerase chain reaction (PCR) and ELISA.

## 4. Molecular biology detection methods

### 4.1. Reverse transcription PCR

Reverse transcription PCR (RT-PCR) is a practical technique that can greatly increase the amount of RNA in trace amounts (Zhu et al., 2020). In the past decade, many researchers have developed novel detection methods based on the single-PCR principle to improve the accuracy and sensitivity of assays based on molecular biology techniques. These more recent detection methods include dual PCR, one-step multiplex PCR, real-time fluorescence quantitative PCR, nested PCR, reverse transcriptase PCR restriction fragment length polymorphism (RT-PCR-RFLP), and other related assays (Hu et al., 2011b). RT-PCR has the following advantages: (i) diagnosis of pathogens present in secretions, tissue samples, blood samples, and cell cultures; (ii) superiority in diagnosing and differentiating different strains of PRRSV; and (iii) compared with VI, RT-PCR has a rapid diagnostic time and can produce results quickly, which can reduce the serious economic losses that an outbreak may cause.

Based on available reports, RT-PCR can be classified into three types, namely, PRRSV general, PRRSV typing discrimination, and discrimination between PRRSV and other viruses. The RT-PCR developed by Zhang et al. (2011) was generalized to detect PRRSV-2. Additionally, the majority of the PRRSV vaccines used in China for prevention and control are highly pathogenic attenuated vaccines. There might be many wild strains or vaccine strains in the same pig herd due to the extensive use of vaccinations on individual farms and its insufficient protection against the present common strains. This has the potential for viral recombination of wild strains with vaccine strains, wild strains with wild strains, and vaccine strains with vaccine strains, which can easily lead to the formation of many new genetic subtypes and novel strains (Wang et al., 2012; Zhao et al., 2015). New strains emerging will only make the outbreak more complex. Therefore, one of the keys to preventing and controlling PRRSV epidemics is to accurately identify whether a classic, highly pathogenic or vaccine strain is prevalent in the pig herd. RT-PCR assays developed by Shi et al. (2012) and Lou et al. (2000) can differentiate PRRSV-1 from PRRSV-2. The RT-PCR assays developed by Hao et al. (2007) and Yang et al. (2013) can differentiate HP-PRRSV from classical PRRSV (C-PRRSV). The assay of Yang et al. (2013) is not only rapid (it can be completed within 2 h), but it can also detect RNA 2 d before the appearance of clinical symptoms, which helps to detect HP-PRRSV and C-PRRSV in advance. The sensitivity detection limit is 25 copies/μL, which is relatively high. Multiplex RT-PCR developed by Yang K. et al. (2017) and Shi et al. (2017) can distinguish between C-PRRSV, HP-PRRSV, and vaccine strains, with the former targeting the HP-PRRSV JXA1-R weak vaccine strain and the latter the TJM-F92 vaccine strain. Analysis of changes in the prevalence of PRRSV indicates that NADC30-like PRRSV is one of the major prevalent strains and has an increasing prevalence in most areas of China. The commonly used HP-PRRSV vaccines have poor cross-protection against NADC30-like PRRSV (Wu et al., 2021; Zhao et al., 2022). The RT-PCR assays developed by Xu and Zhu (2019), Rao (2020), and Wang M. et al. (2022) are able to detect NADC30-like PRRSV to essentially solve the above situation. The detection rate of NADC34-like PRRSV as a potentially prevalent strain is increasing. However, there are no relevant reports on PCR for the diagnosis of NADC34-like PRRSV.

PRRS is a serious immunosuppressive disease that can cause abnormal innate immune cellular immunity and humoral response by affecting thymocyte development, resulting in high susceptibility to secondary or mixed infections (Wang et al., 2020). In general, the occurrence of mixed swine viral infections causes very similar clinical signs. Pathogen differentiation is required for accurate diagnosis. Multiplex PCR assays for clinical diagnosis offer significant advantages, allowing the simultaneous amplification of several viruses in a single reaction mixture, thus facilitating cost-effective diagnosis. The multiplex RT-PCR assays developed by Gu et al. (2019a), Zhou et al. (2019), and Xiang et al. (2022) are able to rapidly detect cases of mixed infections with PRRSV. However, single-specific PCR assays necessitate individual amplification of each target, which is not only costly but also time-consuming. Despite the great diagnostic potential of RT-PCR in PRRSV, the successful development of each RT-PCR assay relies on the content of the nucleic acid-containing samples used for amplification and the precise design of specific primers. At the same time, these RT-PCR assays are open-tube operations with agarose gel electrophoresis detection, which are prone to the risk of PCR product contamination, resulting in inaccurate results. In addition, when new mutant strains emerge, multiplex RT-PCR assays are also unable to perform a differential diagnosis.

### 4.2. Quantitative real-time PCR

Quantitative real-time PCR (qPCR) is a technique performed in a “closed-tube” system for detecting and quantifying gene expression in real time (Singh and Roy-Chowdhuri, 2016). It is based on continuous measurements of the accumulation or reduction of the fluorescence signal during the amplification reaction (Zhu et al., 2020). High-throughput screening can be performed despite low virus levels, and it can be widely used for virus diagnosis (Hawkins and Guest, 2017). qPCR mainly includes the SYBR Green qPCR assay, Eva Green qPCR assay, and Taq Man qPCR assay.

The ReTi RT-PCR assay developed by Martínez et al. (2008) using the SYBR green fluorescent dye is able to differentiate between PRRSV-1 and PRRSV-2. The SYBR Green RT-qPCR assay developed by Chai et al. (2013)is able to distinguish between HP-PRRSV and C-PRRSV, but it cannot detect PRRSV-1 or co-infection with HP-PRRSV and C-PRRSV. Zheng et al. (2020) developed a reliable SYBR Green-based real-time PCR assay for investigating co-infection with PRRSV and porcine circovirus-3 (PCV-3). Meanwhile, SYBR Green RT-PCR is considered a flexible assay that can be applied directly to any gene without the need to design and synthesize fluorescently labeled target-specific probes. The SYBR Green qPCR assay has a lower cost compared with Taq Man’s qPCR (Arvidsson et al., 2008; Khan et al., 2011). Compared with SYBR Green I, the specific steps of the assays based on Eva Green are less prone to non-specific amplification and have a less inhibitory effect on amplification (Khan et al., 2011). Zheng et al. (2017) developed an Eva Green qPCR assay based on melting curve analysis for the detection of PRRSV. The new saturated dye Eva Green used in this study has a strong qualitative and fluorescent signal that is suitable for the development of multiplex PCR assays. At the same time, it compensates well for the common “dye redistribution” in the melting curve of SYBR Green I dye (Cheng et al., 2013). Dye-based RT-qPCR is a qualitative assay, but it suffers from an inability to calculate viral load and is susceptible to false-negatives.

In general, TaqMan probes are specific to SYBR Green and Eva Green dyes and can enhance the identification of true positives. Furthermore, most real-time assays to date have been based on the use of target-specific TaqMan probes. Currently, the PRRSV TaqMan RT-qPCR assay uses specific primers and probes for different genes, such as *Nsp2*, *ORF5*, and *ORF7*. The *Nsp2* coding region has natural point mutations and deletions and is the most variable region in the whole genome. Therefore, *Nsp2* is often used as an important target for the PRRSV species assay (Murtaugh et al., 1995; Cha et al., 2004; Shi et al., 2010). The qPCR assays developed by Qiu et al. (2019), Wang et al. (n.d.), Yu et al. (2022), and Liu et al. (n.d.) were designed with specific primers and TaqMan fluorescent probes based on *Nsp2* to enable rapid detection of PRRSV in mixed infection cases or for differentiation of PRRSV types. The GP5 protein is encoded by *ORF5* and is one of the mutation-prone structural proteins in PRRSV. The *ORF5* gene reflects the genetic variation of PRRSV, which is also the main basis of PRRSV genomic typing (Chen L. et al., 2020). Zhang et al. (2022) developed a universal TaqMan-qPCR assay designed with specific primers and probes based on the conserved sequence of the *ORF5* gene to achieve a comprehensive screening of pig farms. The primers and probes in the PRRSV RT-qPCR assay developed by Song et al. (2006), Chen P. et al. (2021), and Zhang et al. (2022) were designed to be positioned inside the highly conserved gene fragment *ORF7* of PRRSV.

The TaqMan-qPCR assays were developed to provide precise quantification of the number of copies of various virus strains, which serves as a foundation for genetic variation investigation. The RT-qPCR is a closed-tube operation, avoiding the open-tube operation of the agarose gel electrophoresis assay in the RT-PCR assay, reducing the risk of laboratory aerosol pollution, and greatly saving assay time. In addition, the specificity of RT-qPCR is guaranteed by both the probe and primer, making the assay results more reliable. However, TaqMan qPCR also has shortcomings, including a high cost and probes that are very sensitive to sequence changes. Mutations that may be present within the probe binding site can affect the results of probe-based assays. Such mutations can be detrimental to the annealing temperature of the probe and subsequent detection (Martínez et al., 2008). Meanwhile, the gene sequence of PRRSV is very susceptible to mutation and recombination. Therefore, it is critical to design and screen for appropriate probes (Song et al., 2006). Of concern is that the results of qPCR depend on the relationship between the cycling threshold (Ct) and standard calibration curve. The Ct threshold used to quantify the target copy number is subjective, which leads to error amplification that limits its usefulness (Bustin et al., 2005). Conventional PCR and qPCR assays require sophisticated equipment and are not suitable for rapid on-site diagnosis in field situations or in poorly equipped laboratories (Table 2).

**Table 2:** Comparison of different PRRSV qPCR methods.

Types of detection method	Probe/primer design	Specificity	Experimental costs	Characteristics
SYBR Green qPCR Eva Green qPCR	Primer	Medium	Medium	Advantages: flexibility to apply to any gene; no need to design target-specific probes. Disadvantages: inability to calculate viral load; susceptibility to false negative rates.
Taq Man qPCR	Probe + primer	High	High	Advantages: accurate quantification of virus copy numbers of different strains; double assurance with probes and primers. Disadvantages: high cost; probes that are very sensitive to sequence changes; mutations in probe binding sites.

### 4.3. Digital PCR

Digital PCR (dPCR) is an emerging high-throughput detection technique (Pinheiro et al., 2012). The main advantages over conventional PCR and qPCR include: (i) accurate detection and quantification of PRRSV without relying on calibration curves and reaction efficiency and Ct; (ii) detection of PRRSV with greater sensitivity and precision of several orders of magnitude, which can be used for high-throughput detection of multiple infections in clinical samples, especially those targeting very low viral concentrations (Hindson et al., 2011); and (iii) improved tolerance to PCR inhibitors, thereby improving the accuracy of quantification. Yang Q. et al. (2017) developed a dPCR system capable of sensitive and accurate quantification of PRRSV. Shi et al. developed multiplex crystal dPCR for differential detection and quantification of African swine fever virus, classical swine fever virus, and PRRSV (Shi et al., 2022). Because of the very low template concentration, samples that tested negative by multiplex qPCR were positive by the above two assays Therefore, we can consider dPCR for the detection of suspicious samples with negative results using RT-qPCR. At present, reducing the cost of developing dPCR-based PRRSV assays remains a great challenge.

### 4.4. Loop-mediated isothermal amplification

Loop-mediated isothermal amplification (LAMP) is a nucleic acid amplification technique invented by Notomi et al. (2000). This assay designs and synthesizes 4–6 specific primers based on 4–6 regions of the target gene, which has the advantage of high specificity. Under isothermal conditions, it employs a DNA polymerase with a strand substitution function to specifically amplify the target sequence. Because of its low cost, LAMP can be used to detect a wide variety of pathogens (Wang et al., 2019). LAMP is a one-step assay and does not require complex equipment or more cumbersome reaction procedures, which helps to reduce the risk of contamination. Moreover, a technique like LAMP does not require technical personnel and high-end equipment. It can be used in well-equipped laboratories as well as in simple veterinary quarantine departments, providing a simple method for rapid diagnosis of PRRS in the field. Currently, LAMP PRRSV assays have been developed to determine amplification results using gel electrophoresis, real-time turbidity monitoring (obtained from phosphate precipitates), and fluorescent dye-mediated colorimetric assays (Li et al., 2009; Qin et al., 2009; Rovira et al., 2009; Chen J. et al., 2010; Zhang et al., 2011; Gou et al., 2014). LAMP assays based on the addition of a fluorescence or metal indicator to the pre-reaction solution allow for visual interpretation of the assay results based on colorimetric assays. Commonly used fluorescent dyes or metal indicators for the LAMP PRRSV assay include SYBR Green I, PicoGreen, and hydroxynaphthol blue (Qin et al., 2009; Park et al., 2016; Sun et al., 2018; Wang et al., 2019). Notably, during the PRRSV LAMP assay, the positive amplification results after agarose gel electrophoresis show a stepwise pattern, which is not the same as the amplification of specific fragments by conventional PCR (Chen C. et al., 2010; Chen J. et al., 2010). Zhang et al. (2011) used a Loopamp real-time turbidimeter to quantify sensitivity and specificity, which simplified the LAMP detection procedure and improved the efficiency of PRRSV-2 detection. The LAMP method developed by Rovira et al. (2009) and Park et al. (2016) can be used to differentiate between PRRSV-1 and PRRSV-2. The limit of detection of the former is between 102 and 104 TCID_50_/mL, which is significantly lower than that of conventional RT-PCR; in the latter method, the limits of detection are 1 or 0.1 TCID_50_/0.1 ml for PRRSV-1 and PRRSV-2, respectively. Thus, the sensitivity of the assay of Park et al. is superior. However, multiple primer pairs are used in LAMP assays, and dimers are easily formed between primers, resulting in false positives (Wernike et al., 2012). Mutations in the viral primer binding region can affect the accuracy of LAMP. Therefore, mutation sites need to be avoided when designing primers. Many LAMP-based assays have few commercial technologies owing to the cross-reactivity and lack of sensitivity (Rai et al., 2021).

### 4.5. Recombinase polymerase amplification

Recombinase polymerase amplification (RPA) is a new thermostatic amplification technique with a short running time; it is a type of point-of-care testing (Piepenburg et al., 2006). Instant detection technology is a new class of rapid detection technology that has emerged in recent years. RPA is characterized by easy operation and portable equipment; it does not depend on laboratory facilities; and it has great potential in meeting the needs of rapid identification and field diagnosis of animal viruses. In contrast with conventional PCR, RPA does not require an initial denaturation and a cycling step at 95°C. Its experimental procedure is carried out at a constant temperature. Secondly, the RPA reagent is in the form of lyophilized pellets, which do not lose activity easily. Its shelf life at room temperature is up to 12 weeks (Lillis et al., 2016). Therefore, it can be preserved for a long time without a cold chain. In addition, qPCR-specific probes can lead to unsatisfactory experimental results if they fail, whereas synthetic RPA primers and probes with 5–9 mismatches have no effect on assay performance (Abd El Wahed et al., 2013; Boyle et al., 2013). It is also worth noting that the reaction time of RT-RPA is 5–20 min, which is much faster than most assays (Daher et al., 2016). RPA requires low-cost detection equipment and can even be used to detect samples at human body temperature, making it very suitable for field sample detection. At present, the main detection assays developed based on the RPA principle include gel electrophoresis, quantitative fluorescence RPA, RPA-lateral flow dipstick (RPA-LFD), and dye assay. The products after RPA amplification can be detected by agar gel electrophoresis. Compared with normal PCR, the detection speed and sensitivity of RPA are much faster. Many researchers have combined RPA with specific fluorescent probes to develop portable assays that can be used for the rapid detection of nucleic acids with specific, accurate, and sensitive reactions. Yang et al. (2016) and Wang J. et al. (2017) developed an RPA PRRSV assay based on a fluorescent probe. Wang et al. used a fluorescent probe and a Genie III (OptiGene Limited, West Sussex, United Kingdom) tube scanner to measure the amplification products in real time. Additionally, Genie III has the benefit of being easy to carry because of its small size and light weight. Genie III has a built-in rechargeable battery that can support all-day operation. This is especially important for pig farms located in remote areas. RPA combined with a lateral flow dipstick forms the PRRSV RPA-LFD assay (Zeng et al., 2022). Amplification products that react for 20 min at 37°C are mixed with exclusive buffer and then inserted into LFD test strips. Then, 5 min later, the detection results can be observed visually. The assay does not rely on laboratory-specific equipment, but the purchased LFD test strips increase the cost of the experimental fee. There is still no software dedicated to designing RPA primers and probes. The length of its primers and probes is long, which makes the design process more difficult.

### 4.6. Clustered regularly interspaced short palindromic repeats

Clustered regularly interspaced short palindromic repeat (CRISPR) directs RNA to bind to the target complementary sequence, while the nuclease cleaves it at the precise site. Then, specific CRISPR-associated nuclei have “incidental cleavage” activity. For example, cas9, cas12, or cas13 can be used to cleave the target molecule (Paul and Montoya, 2020). Many researchers have tried to use CRISPR-based detection systems in combination with RPA to detect PRRSV, making CRISPR detection more specific (Chang et al., 2020; Liu et al., 2021). CRISPR can also be combined with LFD for a visual readout, making it suitable for use in the field. Moreover, CRISPR assays are designed and performed at a constant temperature, making them easy to use in ill-equipped laboratories or in the field. Based on cas12a combined with RT-RPA amplification, Liu et al. (2021) developed a PRRSV assay with single-stranded DNA-fluorescence quenching (ssDNA-FQ) reporter gene optical features. The assay can complete a thermostatic visualization assay in a single reaction tube within 25 min. Compared with normal PCR and CRISPR/cas12a combination PCR-based assays, CRISPR/cas12a combined with RPA is more sensitive. The sensitivity limit can reach one copy. Although these advanced molecular diagnostic assays have shown excellent results, it is important to carefully validate these tools for effective application in the field.

### 4.7. Metagenomic next-generation sequencing

Currently, sequencing assays have been successfully applied in various fields of virology, including whole virus genome sequencing, the discovery of new viruses and strains, epidemiological investigations, and others (Houldcroft et al., 2017; Gu et al., 2019b). Metagenomic next-generation sequencing (mNGS) is a high-throughput diagnostic tool, primarily used for the unbiased identification of pathogens. It can be divided into two types: amplicon sequencing and whole genome sequencing. When new viruses or mutant strains emerge, diagnostic tools such as RT-PCR require assumptions about the source of infection and do not immediately identify new pathogens. In contrast, next-generation sequencing technology offers enormous advantages. mNGS can identify new pathogens in addition to known pathogens (Chen S. et al., 2020). This is particularly important for a rapid response to highly variable pathogens, such as PRRSV. Nanopore, Sanger, and Illumina sequencing may all successfully identify the presence of PRRSV in samples. With the advantage of excellent accuracy for raw sequences, Illumina, based on short-read sequencing, has the highest throughput of any sequencer on the market (Gu et al., 2019a,b). The whole PRRSV genome may be sequenced with greater than 99.9% accuracy using both Illumina and Sanger sequencing. However, before sequencing, RNA must be converted into cDNA and amplified using PCR for both Illumina and Sanger sequencing (Zhang J. et al., 2017; Han et al., 2019). The nanopore MinION sequencer allows direct sequencing of RNA strands to detect PRRSV, a feature that avoids the bias introduced by PCR. The distinguishing feature of nanopore sequencing is the ability to perform ultra-long reads. By analyzing sufficient sequencing data, the nanopore MinION sequencer has the potential to identify unknown pathogens in the original sample within 1 day. This advantage of real-time sequencing reads makes nanopore sequencing ideally suited for pathogen identification and characterization (Jain et al., 2016). Tan et al. (2019) directly sequenced PRRSV RNA using the Oxford Nanopore MinION sequencer and successfully assembled an almost complete PRRSV genome from raw sequence reads. Direct RNA sequencing reliably detected PRRSV with 99.9% accuracy using only five raw sequencing reads and successfully distinguished multiple strains coexisting in a single sample. Chen et al. (2022) used nanopore sequences in combination with different *de novo* assemblers and polishers to obtain a near-complete PRRSV genome with 99.9% homology. However, the nanopore method is currently prone to sequencing errors and has lower throughput and higher read costs than other NGS platforms, which may limit its usefulness in certain applications (Table 3).

**Table 3:** Comparison of the major molecular biology methods of PRRSV detection.

Types of detection method	Amplification temperature conditions	Characteristics
PCR	Variable	PCR is widely used in PRRSV detection and has been widely accepted by researchers. However, the process requires a variety of biochemical reagents, sophisticated instruments, and trained professionals.
LAMP	Constant	LAMP has the advantage of high specificity by designing 4–6 specific primers based on 4–6 regions of the target gene. However, multiple primer pairs are used in the LAMP, and dimers are easily formed between primers, resulting in false positives.
RPA	Constant	The response time of RPA is much shorter than most detection methods. RPA has low requirements for assay equipment, and it is well suited to on-site sample detection. However, due to the long length of its primers and probes, there are some difficulties in the design process.
CRISPR	Constant	Special CRISPR-associated nucleases have “incidental cleavage” activity and can be used to cleave target molecules. However, it is important to carefully validate these tools for effective application in the field.
mNGS	/	Along with recognized pathogens, mNGS can detect novel pathogens.

## 5. Conclusion

PRRS is one of the most common and economically important swine infectious diseases worldwide. A range of nucleic acid and antigen/antibody-based assays are currently available for the detection of PRRSV. In the face of different situations, the choice of detection method varies. Nucleic acid testing can be used for diagnostic purposes, and antibody testing can be used to assess PRRSV exposure or for herd serum monitoring. VI is required when large quantities of PRRSV strains are needed to develop new assays. At the same time, VI can be used for the identification of new outbreak areas and the confirmation of acute cases. ELISA is the best choice when dynamic patterns of PRRSV antibody elongation are needed to assess whether vaccination has produced an immune response. ELISA can be used not only to confirm early PRRSV infection but also to differentiate between wild strains and vaccine-induced antibodies. IFA and IPMA require the use of microscopes for observation and interpretation of results. The low cost of reagents and consumables required for their experiments makes them particularly suitable for use in primary units. When mixed infections between PRRSV subtypes or between PRRSV and other viruses occur, some newly developed PCR-based PRRSV assays and LAMP assays are available to address these challenges. When there is a very low concentration of the PRRSV template, qPCR, dPCR, and other detection assays can be used, which makes identification more reliable. When new viruses or mutant strains emerge, mNGS, which can identify new strains, is a powerful tool.

Notably, when two assays are used in combination, the advantages of both can complement each other, greatly improving sensitivity and specificity. Comparing the development of each type of research, there is a clear trend toward the use of assays based on molecular biology and bioinformatics. Improving these methods may be a priority for the future. Researchers may also focus on molecular biology and bioinformatics when developing new methods. However, they also suffer from high cost and difficulty in designing probes or primers with strong specificity. The genetic diversity and highly variable recombinant nature of PRRSV has led to the emergence of new strains, which makes the diagnosis of PRRS more difficult. Therefore, the establishment of an accurate, rapid, sensitive, high-throughput, and low-cost assay for PRRSV detection in the future is crucial for decontamination of swine farms.

## Author contributions

JP collected data and wrote the original draft; MZeng complemented and improved the manuscript; MZhao and LH made the final revision of the manuscript. All authors read and approved the final manuscript.

## Funding

This work received funds from the National Natural Science Foundation of China (31902279). The funder had no role in the study design and collection, analysis, and interpretation of the results.

## Conflict of interest

The authors declare that the research was conducted in the absence of any commercial or financial relationships that could be construed as a potential conflict of interest.

## Publisher’s note

All claims expressed in this article are solely those of the authors and do not necessarily represent those of their affiliated organizations, or those of the publisher, the editors and the reviewers. Any product that may be evaluated in this article, or claim that may be made by its manufacturer, is not guaranteed or endorsed by the publisher.

## Supplementary material

The Supplementary material for this article can be found online at:

https://www.frontiersin.org/articles/10.3389/fmicb.2023.1097905/full#supplementary-material
